# Post‐exercise neural plasticity is augmented by adding blood flow restriction during low work rate arm cycling

**DOI:** 10.1113/EP092113

**Published:** 2025-01-21

**Authors:** Mikaela L. Frechette, Summer B. Cook, Brendan R. Scott, Jane Tan, Ann‐Maree Vallence

**Affiliations:** ^1^ Department of Kinesiology University of New Hampshire Durham New Hampshire USA; ^2^ PHysical Activity, Sport and Exercise (PHASE) Research Group School of Allied Health (Exercise Science), Murdoch University Perth Australia; ^3^ Centre for Healthy Ageing, Health Futures Institute Murdoch University Perth Australia; ^4^ School of Psychology, College of Health and Education Murdoch University Perth Australia

**Keywords:** blood flow restriction, exercise intervention, hypoxia, inhibition, motor cortex excitability, neural plasticity

## Abstract

Blood flow restriction (BFR) combined with low work rate exercise can enhance muscular and cardiovascular fitness. However, whether neural mechanisms mediate these enhancements remains unknown. This study examined changes in corticospinal excitability and motor cortical inhibition following arm cycle ergometry with and without BFR. Twelve healthy males (24 ± 4 years) completed four, randomized 15‐min arm cycling conditions: high work rate (HW: 60% maximal power output), low work rate (LW: 30% maximal power output), low work rate with BFR (LW‐BFR) and BFR without exercise (BFR‐only). For BFR conditions, cuffs were applied around the upper arm and inflated to 70% of arterial occlusion pressure continuously during exercise. Single‐pulse transcranial magnetic stimulation was delivered to left primary motor cortex (M1) to elicit motor‐evoked potentials (MEP) in the right biceps brachii during a low‐level isometric contraction. MEP amplitude and cortical silent period (cSP) duration were measured before and 1, 10 and 15 min post‐exercise. MEP amplitude increased significantly from baseline to Post‐10 and Post‐15 for both the HW (both *z *< −7.07, both *P *< 0.001) and LW‐BFR conditions (both *z *< −5.56, both *P *< 0.001). For the LW condition without BFR, MEP amplitude increased significantly from baseline to Post‐10 (*z *= −3.53, *P *= 0.003) but not Post‐15 (*z = *−1.85, *P *= 0.388). The current findings show that HW arm cycling and LW‐BFR led to longer‐lasting increases in corticospinal excitability than LW arm cycling alone. Future research should examine whether the increased corticospinal excitability is associated with the improvements in muscle strength observed with BFR exercise. A mechanistic understanding of BFR exercise improvement could guide BFR interventions in clinical populations.

## Abstract (highlights)

- **What is the central question of this study?**
  Does low work rate arm cycling with blood flow restriction (BFR) lead to an increase in corticospinal excitability and decrease in cortical inhibition that is comparable to high work rate arm cycling and greater than low work rate arm cycling and BFR without exercise?
- **What is the main finding and its importance?**
  Unrestricted high work rate arm cycling and low work rate arm cycling combined with BFR led to longer lasting increases in corticospinal excitability than low work rate arm cycling alone. Future research is needed to examine whether the increased corticospinal excitability is associated with the improvements in muscle strength observed with BFR exercise.

## 1 INTRODUCTION

Blood flow restriction (BFR) combined with low‐load resistance training is an effective strategy for increasing muscle size and strength without needing to lift heavy loads (Lixandrão et al., 2018). Applying BFR during low work rate aerobic training has also been shown to improve cardiovascular fitness (Silva et al., 2019) and functional abilities in the context of rehabilitation (Hedt et al., 2022). As such, BFR is being increasingly promoted as a method to enhance muscular and cardiovascular fitness in individuals who are unable to tolerate the mechanical demands of strenuous exercise, such as those suffering from disease (Corrêa et al., 2021), recovering from injury (Cognetti et al., 2022) and older adults (Centner et al., 2019). This technique involves inflatable cuffs being applied proximally around the exercising limbs, with the aim to partially limit arterial inflow and mostly occlude venous return from the working musculature during exercise (Scott et al., 2015). Interestingly, research has shown that adding BFR during low work rate aerobic training such as cycling can improve muscle strength even in healthy young males and females (de Oliveira et al., 2016; Ozaki et al., 2011), which normally requires dedicated resistance training. This could represent a composite training method to benefit both cardiovascular and muscular fitness. There is some evidence that BFR can augment intracellular signalling for muscle hypertrophy during low work rate aerobic exercise (Ozaki et al., 2014). However, while neural adaptations likely contribute to improvements in strength following traditional resistance training (Siddique et al., 2020), it is unclear whether neural mechanisms may also underpin improvements in muscle strength from low work rate BFR aerobic exercise.

Low‐load BFR resistance exercise has been shown to acutely increase muscle activation, as measured via surface electromyography (EMG) (Yasuda et al., 2009). Based on Henneman's size principle, recruitment of motor units is known to correspond with heightened muscle force production (Henneman et al., 1965). It is plausible that acutely increased muscle activity during training could partly explain the effects of BFR on improving strength. Such neural adaptations may result from changes in corticomotor excitability and inhibition following a bout of BFR exercise, which can be measured via transcranial magnetic stimulation (TMS). This is a non‐invasive technique that can be used to stimulate the primary motor cortex (M1), with a single suprathreshold intensity test pulse eliciting a motor‐evoked potential (MEP) in the muscle(s) controlled by the cortical representation(s) over which the pulse was delivered. The size of the MEP provides a marker of corticospinal excitability and increased corticospinal excitability following exercise is indicative of neural plasticity (Hallett, 2000). GABAergic inhibition plays an important role in neural plasticity (Sanes & Donoghue, 2000). The cortical silent period (cSP) is a period of inactivity in the electromyogram (EMG) from a voluntarily contracted muscle following a suprathreshold TMS pulse (Chen et al., 1999; Fuhr et al., 1991; Inghilleri et al., 1993); the later component of the cSP (50–200 ms) is mediated by cortical GABAergic inhibition (GABA_A_ and GABA_B_ receptor activity: McDonnell et al., 2006; Paulus et al., 2008; Stetkarova & Kofler, 2013; Ziemann et al., 2015). Thus, it is plausible that exercise‐induced neural plasticity, mediated via a reduction in GABAergic inhibition, could underlie strength improvements observed with BFR.

Brandner et al. (2015) used TMS to investigate corticospinal excitability following acute upper body resistance exercise sessions with BFR. The MEP amplitude increased from baseline following low‐load resistance exercise (20% 1‐repetition maximum) and remained elevated for 20 min in a no‐BFR condition, 40 min if cuffs were applied intermittently (i.e., deflated between sets), and for at least 60 min when cuffs were inflated continuously (i.e., not deflated between sets). Furthermore, the continuous BFR condition resulted in significantly higher MEP amplitudes from 5–60 min post‐exercise than all other exercise trials (including high‐load resistance exercise). These results suggest an acute neuromuscular mechanism by which continuous BFR application could underpin strength improvements following upper body resistance training. It is possible that the same results may be observed for low work rate aerobic exercise with BFR, though this is yet to be investigated.

The acute increased muscle activation (Yasuda et al., 2009) and corticospinal excitability (Brandner et al., 2015) observed when BFR is applied during low‐load resistance exercise may be related to improvements in strength resulting from multiple exposures across a training programme (Centner & Lauber, 2020). However, these acute neuromuscular responses have not yet been investigated for aerobic BFR exercise, which limits our mechanistic understanding of how aerobic BFR training may improve muscle strength. Therefore, the purpose of the current study was to compare whether low work rate arm cycling with BFR leads to an increase in corticospinal excitability and decrease in cortical inhibition that is comparable to high work rate arm cycling, and greater than low work rate arm cycling and BFR without exercise. It was hypothesized exercise‐induced changes in MEP amplitude and cSP would be greater following high work rate arm cycling and low work rate arm cycling with BFR compared to low work rate arm cycling.

## 2 METHODS

### 2.1 Ethical approval

All participants provided signed, written informed consent. The experiment conformed to the standards set by the latest revision of the *Declaration of Helsinki*. The experimental procedures were approved by Murdoch University's Human Research Ethics Committee (2017‐046) and the University of New Hampshire's Institutional Review Board (2017‐6672).

### 2.2 Participants

Measures of MEP amplitude and cSP obtained here were part of a larger study examining physiological responses following arm cycling with BFR (Frechette et al., 2023). Twelve recreationally active male university students (age: 24±4 years), who were novices to arm cycling, were recruited for this study. Based on pre‐screening questionnaires, any individual with a personal history of cardiovascular complications, neurological diseases, disorders or trauma, had an inserted medical device, were on medications acting on the central nervous system, or had conditions that would contraindicate TMS (Rossi et al., 2009, 2011) were excluded from the study. Right hand dominant participants were recruited for this study as limb dominance might affect arterial occlusion pressure (Tafuna'i et al., 2021). The Edinburgh Handedness Inventory was used as a screening tool for handedness; no participant scored less than the cut‐off score of 40 used to exclude left‐handed and ambidextrous people (median 83, range 65–100) (Oldfield, 1971).

### 2.3 Experimental design

Participants visited the laboratory for five separate testing sessions. Visit 1 involved familiarization with the research methods and peak oxygen consumption (${\dot{V}}_{O_{2}\mathrm{peak}}$) assessment via a graded exercise test with an arm cycle ergometer. Following familiarization, a within‐subject cross‐over design was used to investigate the acute corticospinal excitability and inhibition, cardiovascular demands and metabolic responses during four experimental conditions in a randomized order: low work rate arm cycling (LW), low work rate arm cycling with BFR (LW‐BFR), high work rate arm cycling (HW) and BFR with no exercise (BFR‐only). Arm cycling was used in this study so that measures of corticospinal excitability and inhibition could be measured from the trained, upper‐limb muscle representations in M1. The cardiovascular and metabolic responses are reported elsewhere (Frechette et al., 2023). Randomization was achieved using an online randomizer (https://www.randomizer.org). All sessions were separated by at least 2 days to allow recovery from exercise (which is sufficient because no muscle damage is expected following the 15 min concentric exercise protocol), and were completed at the same time of day to control for time‐of‐day influences on neuroplasticity (Sale et al., 2007). Participants were instructed to refrain from exercise 24 h before each testing session and not consume any stimulants, such as caffeine, on the day of testing. In all sessions, MEPs elicited by single‐pulse TMS were obtained at baseline, and 1, 10 and 15 min post‐exercise protocol or BFR‐only protocol.

#### 2.3.1 Familiarization (visit 1)

Participants completed a graded, maximal effort arm cycling test and were familiarized with the BFR cuffs and TMS. For the graded arm cycling test, participants were positioned on the arm cycle ergometer (First Degree Fitness E‐820 Fitness Upper‐Body Ergometer, Mandurah, WA, Australia) with their shoulders level with the centre of the ergometer shaft and the elbow joint of the extended arm at 165–175° (Brandner et al., 2015). Participants completed a 3‐min general warm‐up at a power output range of 20–30 W, as measured and provided by the ergometer. This was followed by a 2‐min rest period, during which straps were attached to the participants’ wrists to secure their hands to the ergometer's handles and limit the impact of grip strength on arm cycling performance. The graded protocol began at 60 W, and work rate increased every 2 min by participants cycling 5 revolutions per minute (RPM) faster (equivalent to ∼20 W increase). The test ceased when participants reached volitional exhaustion or their pace was >4 RPM below the target cadence for 15 s.

During the graded arm cycling test, expired gases were measured at baseline, start of exercise, and continuously throughout the exercise protocol using a calibrated Parvo TrueOne metabolic cart (Parvo Medics, East Sandy, Utah), from which the 15 s mean ${\dot{V}}_{O_{2}}$ and respiratory exchange ratio (RER) were calculated. Power output was manually recorded from the arm ergometer at baseline and every 2 min of exercise. Each participant's maximal power output upon reaching ${\dot{V}}_{O_{2}\mathrm{peak}}$ was used to prescribe exercise intensities for each of the exercise conditions tested in separate experimental sessions (described in detail below).

#### 2.3.2 Experimental conditions (visits 2–5)

A 3‐min general warm‐up (power output range of 20–30 W) followed by a 2‐min rest period was completed prior to all experimental conditions, which comprised 15 min of arm cycling. The HW condition was performed at 60% of maximum power output achieved at ${\dot{V}}_{O_{2}\mathrm{peak}}$, and both LW conditions (LW and LW‐BFR) were performed at 30% of maximum power output achieved at ${\dot{V}}_{O_{2}\mathrm{peak}}$. During the BFR‐only condition, the cuff was inflated and participants sat still for 15 min with their hands positioned on the ergometer handles (7.5 min with right arm extended and 7.5 min with left arm extended; a single switch at 7.5 min was to ensure equal time in each posture and minimize movement during BFR). TMS measurements were recorded at baseline and post‐exercise.

### 2.4 Determination and implementation of blood flow restriction pressure

Prior to each BFR condition, participants rested for 5 min and then had their blood pressure measured as per normal procedures and arterial occlusion pressure determined on their left arm in accordance with previous methods (Loenneke et al., 2015). An inflatable cuff (83 cm long, 5 cm wide) was then placed around the most proximal portion of the left arm and connected to an E20 rapid cuff inflator and AG101 air source (Hokanson, Bellevue, WA, USA). During the assessment, blood flow through the radial artery was monitored using a hand‐held bidirectional Doppler ultrasound (M6 Doppler, Hokanson). In the LW‐BFR and BFR‐only conditions, the cuffs were applied to both arms and inflated to 70% of individualized arterial occlusion pressure for the duration of the 15‐min protocol. Based on the results from Mouser et al. (2017), the 5 cm wide cuffs used in our study on the upper limbs would be expected to elicit reductions in blood flow to ∼23% of resting flow rates.

### 2.5 Electromyography

Surface EMG was recorded using Ag/AgCl electrodes positioned according to SENIAM guidelines (Hermens et al., 2000) over the belly of the muscle to record MEPs elicited by TMS. The surface EMG signal was amplified (1000; CED 1902 amplifier, Cambridge Electronic Design (CED), Cambridge, UK), band‐pass filtered (20–1000 Hz), digitized at a sampling rate of 2 kHz (CED 1401 interface), and recorded using Signal 6.02 (CED). At the beginning of each testing session, participants completed a maximal voluntary isometric contraction (MVIC) of the right biceps for 3 s. For all participants, the right arm positioned was standardized at 90° of elbow flexion by holding a secured handle. EMG activity was recorded during the MVIC using the parameters outlined above (the ergometer was not used for MVIC testing).

### 2.6 Transcranial magnetic stimulation

TMS was applied using a Magstim 200^2^ stimulator (Magstim Co. Ltd, Whitland, UK) with a figure‐of‐eight (90 mm diameter) Alpha Flat Coil (Magstim). The TMS coil was placed tangentially to the left M1 with the handle positioned backwards and ∼45° away from the midline to induce posterior–anterior current flow in the cortex.

During all TMS measures, participants sat in a chair with their right arm positioned at 90° of elbow flexion, holding a secure handle, maintaining an isometric hold (5% of MVIC) of the right biceps, consistent with previous research (Brandner et al., 2015). Single pulses (monophasic pulse waveforms) were delivered to the left M1 to elicit MEPs in the right biceps brachii. Suprathreshold pulses were delivered over the left M1 at multiple sites to identify the site from which bicep MEPs were evoked consistently, known as the optimal stimulation site. The optimal stimulation site was marked on the scalp with water‐soluble ink to allow reliable placement of the coil throughout the experimental session. Active motor threshold (AMT) was defined as the minimum stimulus intensity (as a percentage of maximum stimulator output) required to elicit MEPs of at least 0.2 mV in at least 3 of 5 consecutive trials during isometric biceps contraction of 5% of MVIC. The optimal stimulation site and AMT were identified in each session. All measures were obtained by a single assessor.

Before and after the 15‐min exercise or BFR‐only protocol, single‐pulse TMS was applied during isometric biceps contraction of 5% of MVIC at an intensity of 130% AMT. Blocks of 25 stimuli were applied at baseline, 1, 10 and 15 min post‐exercise protocol or BFR‐only protocol. We were interested in characterizing the time course of short‐term changes in cortical excitability following aerobic exercise (with and without BFR) as previous research has shown maximal changes at 20 min following resistance exercise with BFR (Brandner et al., 2015). The cSP is an inhibitory process thought to be mediated by GABA receptors (McDonnell et al., 2006; Paulus et al., 2008; Stetkarova & Kofler, 2013; Ziemann et al., 2015). The cSP duration was quantified as the time between the TMS pulse and the return of baseline EMG activity for all single‐pulse TMS trials.

### 2.7 Data processing

The root mean square of EMG activity was calculated from the 250 ms prior to TMS (pre‐TMS root mean square (RMS) EMG activity) to examine background EMG activity across conditions and time points, as EMG activity during this time frame can influence MEP amplitude. The peak‐to‐peak MEP amplitude was scored from 40 ms of EMG activity beginning 5 ms after TMS application. Mean MEP amplitude was calculated from the 25 trials delivered at each time point for each experimental condition. The cSP duration was scored manually using a custom‐made script (Signal, CED) that displayed raw and rectified EMG traces for each trial: one vertical cursor was automatically positioned at TMS onset, and a second vertical cursor was manually positioned at the EMG activity onset by the experimenter; the time (in ms) between the two vertical cursors was scored as the cSP.

### 2.8 Statistical analysis

All analyses were performed on *n* = 12 using R (version 4.3.2, 2023‐10‐31 ucrt; R Foundation for Statistical Computing, Vienna, Austria) and RStudio (2024.04.2). To test for differences in AMT between conditions, a one‐way ANOVA with the within‐subject factor of condition was performed on AMT data. The Shapiro–Wilk test for normality showed MEP and cSP data were not normally distributed. Generalized linear mixed effect models (GLMMs) were therefore used to analyse MEP and tremor data. As both MEP and cSP data were non‐negative and positively skewed, a gamma distribution with a log link function was applied for each GLMM (Puri & Hinder, 2022). Wald's chi‐square test was conducted for null hypothesis significance testing of main and interaction effects. Significant effects were investigated with Bonferroni‐corrected contrasts. Cohen's *d* effect sizes are reported (*d *< 0.2 representing a small effect, *d* ≥ 0.5 a moderate effect, and *d*  ≥ 0.8 a large effect) (Cohen, 1988). Statistical significance was set at α = 0.05.

Single‐trial‐level MEP amplitudes and cSPs were analysed with separate GLMMs, both with fixed factors of Time (baseline, 1, 10 and 15 min post‐intervention) and Condition (HW, LW, LW‐BFR and BFR‐only), with by‐subject intercept as a random effect. To examine the potential effect of background EMG activity on MEP amplitudes, pre‐TMS RMS was included as a covariate in the GLMM.

To determine whether a change in MEP amplitude post‐exercise was associated with a change in cSP duration post‐exercise, MEP amplitude and cSP duration were normalized to baseline: MEP amplitude at post‐1, post‐10 and post‐15 was expressed as a ratio of MEP amplitude at baseline; cSP duration at post‐1, post‐10 and post‐15 was expressed as a ratio of cSP duration at baseline. Pearson's correlations were performed on the normalized data to assess the relationship between change in MEP amplitudes and change in cSP duration at all post‐intervention time points (*P *< 0.01). Data are presented as means ± SEM unless otherwise noted.

## 3 RESULTS

### 3.1 Baseline TMS characteristics

AMT did not differ across experimental conditions (means and standard deviation presented in Table 1; *n* = 12). A one‐way repeated measures ANOVA showed no main effect of condition for AMT (*F*_3,33_ **= 0.76, *P *= 0.522, η^2 ^= 0.07).

**TABLE 1:** Active motor threshold for each of the four experimental conditions (*n* = 12).

	HW	LW‐BFR	LW	BFR‐only
Active motor threshold (%MSO)	45 (7.2)	44 (5.4)	45 (7.0)	44 (5.8)

Abbreviations: BFR‐only, blood flow restriction with no exercise; HW, high work rate arm cycling; LW, low work rate arm cycling; LW‐BFR, low work rate arm cycling with BFR; MSO, maximal stimulator output.

### 3.2 Change in cortical excitability following arm cycling

**FIGURE 1:**
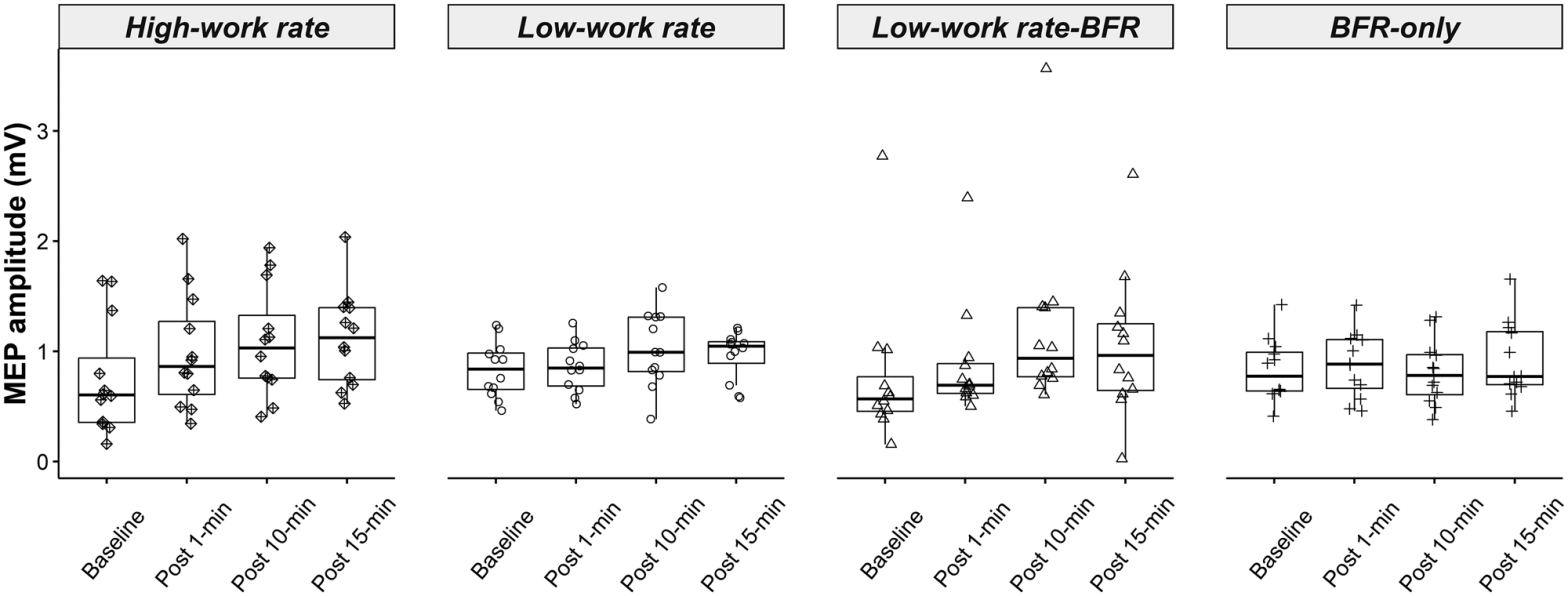
Raw MEP amplitude (in mV) across time for each of the four exercise conditions for all post‐intervention time points (*n* = 12). For both the HW and LW‐BFR conditions, MEP amplitude was significantly increased from baseline to Post‐10 and Post‐15 (all *P *< 0.001); for the LW condition without BFR, MEP amplitude was significantly increased from baseline to Post‐10 (*P *< 0.001) but not Post‐15. BFR, blood flow restriction; HW, high work rate arm cycling; LW, low work rate arm cycling; LW‐BFR, low work rate arm cycling with BFR; MEP, motor‐evoked potentials.

Figure 1 shows changes in MEP amplitude from pre‐ to post‐exercise or BFR‐only for all conditions (*n* = 12). The GLMM analysis on MEP amplitudes found a significant Time × Condition × pre‐TMS RMS interaction (χ^2^ (1, *n* = 12) = 76.87, *P *< 0.001). *Post hoc* analysis showed that for both the HW and LW‐BFR conditions, MEP amplitude was significantly increased from baseline to Post‐10 (HW: *z* = −7.07, *P *< 0.001, *d* = 0.67; LW‐BFR: *z* = −7.54, *P *< 0.001, *d* = 0.69) and Post‐15 (HW: *z* = −9.11, *P *< 0.001, *d* = 0.86; LW‐BFR: *z* = −5.56, *P *< 0.001, *d* = 0.49), but not Post‐1 (HW: *z* = −2.60, *P* = 0.056, *d* = 0.24; LW‐BFR: *z* = −2.00, *P* = 0.272, *d* = 0.18). For the LW condition without BFR, MEP amplitude was significantly increased from baseline to Post‐10 (z = −3.53, *P* = 0.003, *d* = 0.31) but not Post‐15 (*z* = −1.85, *P* = 0.388, *d* = 0.16) or Post‐1 (*z* = −0.09, *P* = 1.000, *d* = 0.01). For the BFR‐only condition, MEP amplitude was not significantly different from baseline at any of the post time points (all *z* > −2.13, all *P *> 0.197, all *d* < 0.20).

### 3.3 Changes in cortical silent period following arm cycling

**FIGURE 2:**
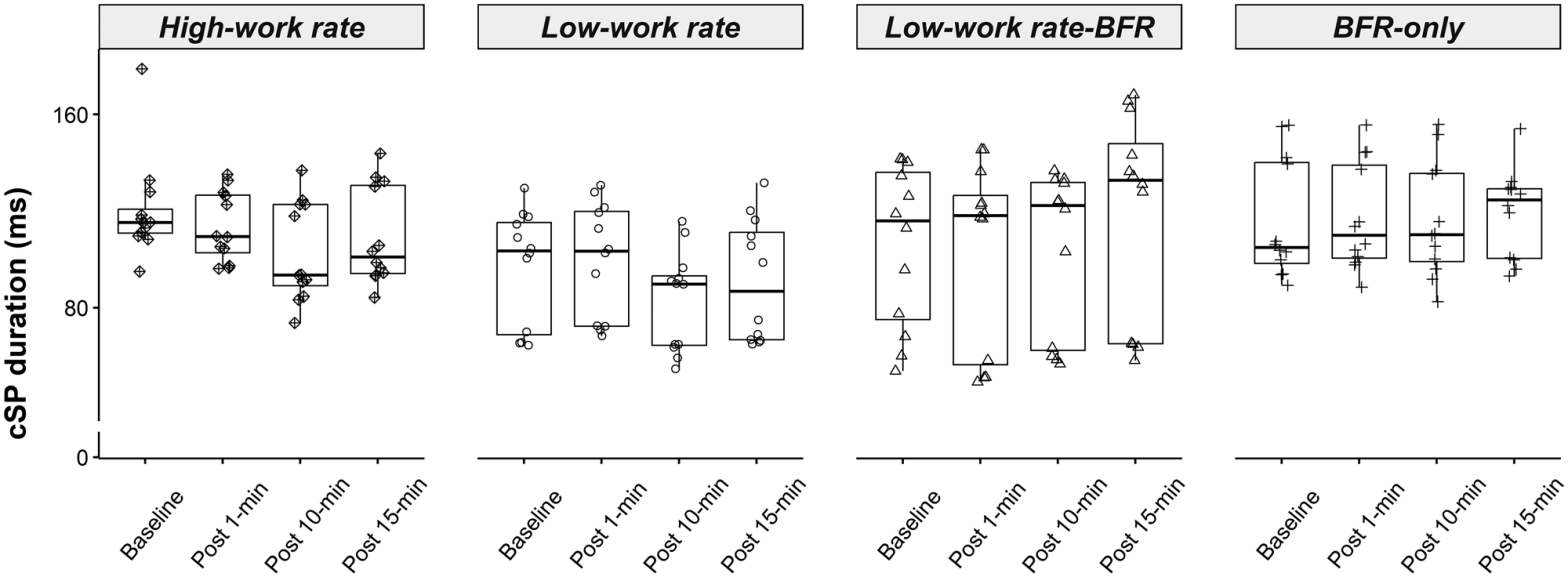
Raw cSP duration (in ms) across time for each of the four exercise conditions for all post‐intervention time points (*n* = 12). For the HW condition, cSP duration was significantly decreased from baseline to Post‐10 and Post‐15 (both *P *< 0.007); for the LW condition without BFR, cSP was significantly decreased from baseline to Post‐10 (*P *< 0.001) but not Post‐15; for the LW‐BFR condition, cSP duration was significantly increased from baseline to Post‐15 (*P *< 0.001) but not Post‐10. BFR, blood flow restriction; cSP, cortical silent period; LW‐BFR, low work rate arm cycling with BFR.

Figure 2 shows changes in cSP duration from pre‐ to post‐exercise or BFR‐only for all conditions (*n* = 12). The GLMM analysis on cSP found a significant Time × Condition interaction (χ^2^ (1, *n* = 12) = 91.34, *P *< 0.001). *Post hoc* analysis showed that for the HW condition, cSP was significantly decreased from baseline to Post‐10 (*z* = 5.95, *P *< 0.001, *d* = 0.56) and Post‐15 (*z* = 3.24, *P* = 0.007, *d* = 0.31) but not at Post‐1 (*z* = 1.00, *P* = 1.000, *d* = 0.10). For the LW condition without BFR, cSP was significantly decreased from baseline to Post‐10 (*z* = 5.92, *P *< 0.001, *d* = 0.52) but not Post‐15 (*z* = 2.08, *P* = 0.224, *d* = 0.18) or Post‐1 (*z* = −1.49, *P* = 0.816, *d* = −0.13). For the LW condition with BFR, cSP was significantly increased from baseline to Post‐15 (*z* = −4.67, *P *< 0.001, *d* = −0.42) but not Post‐1 (*z* = 0.90, *P* = 1.000, *d* = 0.08) or Post‐10 (*z* = 0.252, *P* = 1.000, *d* = 0.02). For the BFR‐only condition, cSP was not significantly different from baseline at any of the post timepoints (all *z* < −1.18, all *P* = 1.000).

### 3.4 Correlation between MEP amplitude and cortical silent period

Pearson's correlations were performed on the change in MEP amplitude and the change in cSP duration following each exercise condition to determine if those individuals who showed the greatest increase in MEP amplitude showed the greatest decrease in cSP duration, as this would indicate that a reduction in GABAergic inhibition mediates the observed increase in MEP amplitude. In the current study, there were no significant associations between normalized MEP amplitudes and normalized cSP data when correcting for multiple comparisons (Table 2), suggesting that the increase in cortical excitability is not mediated by a reduction in GABAergic inhibition.

**TABLE 2:** Pearson's correlations between normalized MEP amplitudes and normalized cSP duration for all conditions (*n* = 12): high work rate (HW), low work rate (LW), low work rate with blood flow restriction (LW‐BFR) and blood flow restriction without exercise (BFR‐only) at time points 1 min post‐exercise (Post‐1), 10 min post‐exercise (Post‐10) and 15 min post‐exercise (Post‐15).

	HW	LW	LW‐BFR	BFR‐only
Post‐1	*r* = 0.014 (*P* = 0.965)	*r* = −0.313 (*P* = 0.321)	*r* = 0.164 (*P* = 0.610)	*r* = −0.126 (*P* = 0.697)
Post‐10	*r* = 0.137 (*P* = 0.672)	*r* = 0.351 (*P* = 0.264)	*r* = 0.012 (*P* = 0.971)	*r* = −0.078 (*P* = 0.809)
Post‐15	*r* = 0.238 (*P* = 0.457)	*r* = 0.058 (*P* = 0.857)	*r* = −0.335 (*P* = 0.287)	*r* = −0.214 (*P* = 0.505)

Abbreviations: BFR‐only, blood flow restriction with no exercise; HW, high work rate arm cycling; LW, low work rate arm cycling; LW‐BFR, low work rate arm cycling with BFR.

### 3.5 Pre‐TMS EMG activity

**FIGURE 3:**
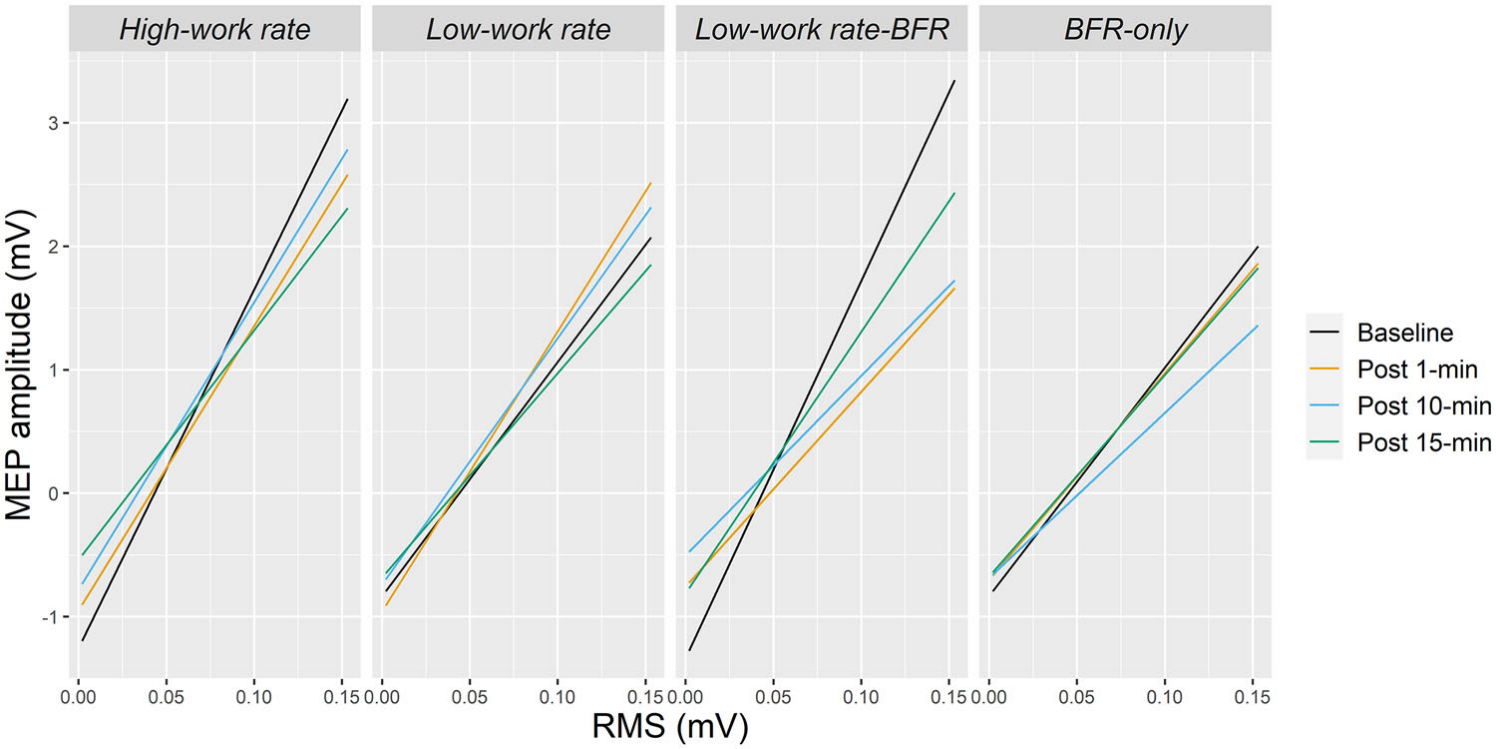
The estimates of the slopes of the pre‐TMS RMS trend for each condition for each time point. In all conditions, as RMS increased, MEP amplitudes increased. MEP, motor‐evoked potentials; RMS, root mean square; TMS, transcranial magnetic stimulation.

Figure 3 shows the estimates of the effects of pre‐TMS RMS as a covariate. For all time points and conditions, MEP amplitudes increased as pre‐TMS RMS increased. For HW, there was a significant difference in pre‐TMS RMS trends between baseline and Post‐15 (|*z*| = 4.40, *P* = 0.012). For LW‐BFR, there was a significant difference in pre‐TMS RMS trends between baseline and all post time points (all |*z*| > 5.40, *P* < 0.001). For LW without BFR and BFR‐only, there were no differences in pre‐TMS RMS trends between baseline and any of the post timepoints (all |*z*| < 2.06, *P* > 0.079).

### 3.6 DISCUSSION

The current study investigated neural changes following aerobic exercise both with and without BFR. The ‘highlights’ from this study were that unrestricted high work rate arm cycling and low work rate arm cycling combined with BFR led to longer lasting increases in corticospinal excitability than low work rate arm cycling alone. Further, 15 min of BFR‐alone did not lead to any changes in corticospinal excitability.

### 3.7 Increased corticospinal excitability following arm cycling

In the current study, MEP amplitude increased for at least 15 min post‐exercise in both HW and LW‐BFR conditions. Following LW, MEP amplitude increased 10 min post‐exercise but returned to baseline levels by 15 min post‐exercise, and there was no change in MEP amplitude following BFR‐only. These results suggest that high and low work rate arm cycling for 15 min can induce an increase in corticospinal excitability, indicative of an exercise‐induced plasticity, but that high work rate exercise leads to a longer‐lasting plastic response than low work rate exercise unless it is coupled with BFR. This is the first study, to our knowledge, to examine changes in corticospinal excitability in the exercised muscle following arm cycling. Several studies have examined corticospinal excitability of the non‐exercised upper‐limb muscles after lower‐limb cycling, and these studies have consistently shown no change in corticospinal excitability of the untrained muscle irrespective of exercise work rate (McDonnell et al., 2013; Mooney et al., 2016; Singh et al., 2014; Smith et al., 2014; Stavrinos & Coxon, 2016). One study measured MEPs in the trained tibialis anterior following lower‐limb cycling, reporting no change in MEP amplitude following 7 min of low work rate exercise (Yamaguchi et al., 2012). Several studies have also examined changes in corticospinal excitability following resistance training, with a meta‐analysis showing no change in corticospinal excitability measured at rest post‐resistance training, but an increase in corticospinal excitability measured during voluntary contraction post‐resistance training (Siddique et al., 2020). One recent study combined resistance training with BFR and showed no change in corticospinal excitability following low‐load leg press with BFR at two different limb occlusion pressures (Norbury et al., 2024). The increased corticospinal excitability following low work rate exercise with BFR in the current study might be due to altered afferent input to the cortex (Brandner et al., 2015). Metabolic changes that occur during BFR, including reduced O_2_ and increased CO_2_, H^+^, and metabolites, affect group III and IV muscle afferents (Yasuda et al., 2010). This change in group III and IV fibres affects afferent input to the M1, which can influence corticospinal excitability (Brasil‐Neto et al., 1992; Gandevia et al., 1996; Ridding & Rothwell, 1995; Vallence et al., 2012). These changes likely underpin, in part, the increased muscle activation during exercise with BFR that is comparable to increased muscle activation during high work rate exercise. It is possible that the increased muscle activation during low work rate exercise with BFR and high work rate exercise (but not low work rate exercise alone) is part of a positive cycle with corticospinal excitability: increased muscle activation leads to an increase in the afferent input to the cortex, which drives increased corticospinal excitability, which in turn, leads to increased muscle activation. The changes in muscle activation and neural drive might underpin the changes in strength observed following exercise with BFR. This, however, remains speculative, and future research should comprehensively measure peripheral changes (i.e., oxygen reduction, gene expression, metabolic byproducts, muscle activation) and neural drive during and following exercise with BFR to understand the interactions between these factors and the association with increased strength. It is important to note, however, that the observed changes in corticospinal excitability following low work rate exercise with BFR could also be due to changes at the spinal level: future research should examine possible changes at the spinal level following high work rate and low work rate with BFR arm cycling.

In addition to showing that 15 min of arm cycling is sufficient to increase corticospinal excitability in the trained muscle representation, the current results show that the increase in corticospinal excitability following low work rate exercise with BFR is comparable to high work rate exercise. This finding is in line with the work of Brandner et al. (2015) showing increased corticospinal excitability (measured during low‐level voluntary contraction of the biceps) in the trained muscle representation following resistance training with BFR (continuous and intermittent bicep curls). However, in contrast to the current results, which showed comparable increases in excitability with high work rate and low work rate with BFR exercise, Brandner et al. (2015) showed that BFR exercise led to greater increases in corticospinal excitability than both heavy and low‐load resistance exercise. Importantly, the two findings strongly suggest that low work rate exercise with BFR leads to physiological and neurophysiological changes that are at least equivalent to high work rate exercise, providing a basis for the use of this exercise protocol in populations who cannot perform high work rate exercise. It is worth noting that MEP amplitude in the high work rate and low work rate with BFR condition did not return to baseline levels 15 min post‐exercise suggesting that corticospinal excitability might remain elevated for a longer duration. It would be interesting to measure MEP amplitude at later time points (up to 60 min post‐exercise) to examine the full time course of changes in corticospinal excitability following high work rate and low work rate with BFR arm cycling.

### 3.8 Changes in cSP duration following arm cycling

In the current study, there were opposing changes in cSP duration following arm cycling with and without BFR: with BFR (LW‐BFRE condition), cSP duration was increased 15 min post‐exercise; without BFR, cSP was decreased 10 and 15 min post‐exercise in the HW condition and 10 min post‐exercise in the LW condition. There was no change in cSP duration in the BFR‐only condition. The opposing changes in cSP duration post‐exercise in the conditions with and without BFR suggest that different mechanisms mediate the induced increases in corticospinal excitability.

The cSP is an inhibitory process, with the early component (<50 ms) thought to be mediated by spinal mechanisms and the later component (50–200 ms) mediated by cortical mechanisms (Chen et al., 1999). GABAergic inhibition is the likely mechanism underlying the cortical component of the cSP, with evidence suggesting a role for both GABA_A_ and GABA_B_ receptor activity (McDonnell et al., 2006; Paulus et al., 2008; Stetkarova & Kofler, 2013; Ziemann et al., 2015). Therefore, the current results suggest that the increase in MEP amplitude observed following exercise without BFR is likely mediated by a reduction in GABAergic inhibition, which is in line with the evidence showing a role of GABAergic inhibition in use‐dependent plasticity and learning (Butefisch et al., 2000; Rogasch et al., 2009). A meta‐analysis of pooled data from studies that examined cSP duration with resistance training protocols indicates that there is a reduction in the duration of the cSP post‐resistance training (Siddique et al., 2020). Several studies have examined the effect of lower‐limb cycling on inhibitory processes acting on the untrained upper‐limb muscles and the results are mixed: most studies show decreased short‐interval intracortical inhibition following exercise (Singh et al., 2014; Smith et al., 2014; Stavrinos & Coxon, 2016), but there is a report of no change in this inhibitory process following exercise (Mooney et al., 2016). Only two studies have examined changes in long‐interval intracortical inhibition—a cortical inhibitory process mediated by GABA_B_ receptor activity (Valls‐Sole et al., 1992; Wassermann et al., 1996)—in the untrained muscle following aerobic exercise, with one showing a decrease (Mooney et al., 2016) and one showing no change in this inhibitory process following exercise (Singh et al., 2014).

In contrast, following exercise with BFR, there was both an increase in cSP duration and an increase in MEP amplitude; as such, cSP cannot be the mechanism mediating the increase in MEP amplitude. Therefore, a reduction in other inhibitory processes or an increase in glutamatergic excitatory processes might mediate the increase in MEP amplitude following exercise with BFR. Brandner et al. (2015) showed no change in short‐interval intracortical inhibition—a cortical inhibitory process mediated by GABA_A_ receptor activity—following exercise with BFR. While at first glance these findings suggest that a reduction in intracortical inhibition is unlikely to mediate the increase in MEP amplitude following arm cycling with BFR, future research systematically and comprehensively investigating the effects of exercise on GABAergic inhibition is warranted. For example, the sensitivity of circuits mediating both short‐interval intracortical inhibition and long‐interval intracortical inhibition can be measured using stimulus–response functions with a range of conditioning stimulus intensity. It would be interesting to determine whether the sensitivity of these circuits is affected by low work rate exercise with BFR. It is also possible that changes in glutamatergic excitatory circuits mediates the exercise‐induced increase in corticospinal excitability, or that changes in the balance between excitation and inhibition are important for exercise‐induced plasticity. Indeed, recent research has shown that high work rate exercise can influence changes in the excitation–inhibition balance induced by non‐invasive brain stimulation protocols (Andrews et al., 2019; Curtin et al., 2023). It would also be interesting to examine potential exercise‐induced changes in the excitability of short‐interval intracortical facilitatory circuits (Ziemann et al., 1998), given their interaction with GABAergic inhibition (Peurala et al., 2008).

### 3.9 Limitations

In the current study, MEP amplitude was not normalized to the maximal compound muscle action potential (*M*_max_). One recent study combined BFR with resistance exercise and showed no change in *M*_max_ following any exercise or BFR conditions (Norbury et al., 2024). However, there is a recent report that V‐wave/M‐wave decreases following BFR with resistance training, with the authors suggesting neuromuscular fatigue at the peripheral and cortical level (Hill et al., 2022). While the current findings show evidence of increased excitability following BFR with aerobic exercise, it remains unknown whether BFR with aerobic exercise induces peripheral changes that are comparable to those seen following BFR with resistance training, or whether changes in peripheral factors (such as muscle fibres) affected MEP amplitude in the current study. Given we cannot rule out changes in peripheral factors (such as muscle fibres), future research should measure *M*_max_ to determine whether changes in muscle fibre activity are observed following low work rate aerobic exercise with BFR. In addition, to probe changes at the spinal level following low work rate aerobic exercise with BFR, future research should measure cervicomedullary evoked potentials (CMEPs). It would also be interesting to fully characterize the time course of changes in cortical excitability following aerobic exercise with BFR. For example, previous research has shown that increases in cortical excitability following resistance training with continuous BFR are maximal at 20 min following exercise and remain for 60 min following exercise (Brandner et al., 2015). Future research should examine cortical excitability for up to 60 min following aerobic exercise with BFR.

In the current study, the analysis performed on MEP amplitudes showed a significant interaction with the covariate pre‐TMS RMS of EMG activity (significant Time × Condition × pre‐TMS RMS interaction). Analyses of the estimates of the effects of pre‐TMS RMS of EMG activity on MEP amplitude showed that MEP amplitudes increased as pre‐TMS RMS increased for all time points and all conditions. While it is known that increased EMG activity is associated with increased MEP amplitude, in the current study, it is unlikely that a change in pre‐TMS RMS post‐exercise underpinned the increase in MEP amplitude post‐exercise: the slope of EMG activity and MEP amplitude was steeper at baseline than the post‐exercise time points for the high work rate and low work rate with BFR conditions, which were the conditions that showed increased MEP amplitude 10 and 15 min post‐exercise. The less steep slope between pre‐TMS RMS of EMG activity and MEP amplitude at the post‐exercise time points suggests that the increase in corticospinal excitability following exercise is due to some other mechanism.

The current sample comprised novices to arm cycling: all participants completed a familiarization session and the order of conditions was randomized; however, we cannot be certain that there were no learning effects. It is also important to note that the current project only tested male participants. Future research should include a larger sample, with equal males and females to comprehensively determine the effect of low work rate aerobic exercise with BFR on corticospinal excitability and inhibition.

### 3.10 Conclusions

Adding BFR to low work rate arm cycling led to a longer‐lasting increase in corticospinal excitability compared to unrestricted low work rate arm cycling. Future research should comprehensively examine associations between increases in neural drive, specifically increased corticospinal output from M1, and the improvements in muscle strength observed with BFR exercise. Further research should also examine other cortical and spinal mechanisms of BFR‐induced improvements in muscle strength. A greater understanding of the mechanisms of BFR exercise improvement could be valuable to guide BFR interventions in clinical populations that might benefit from low work rate exercise with BFR.

## AUTHOR CONTRIBUTIONS

All experiments were performed in the laboratory facilities at Murdoch University, Western Australia. Mikaela Frechette, Summer Cook, Brendan Scott, and Ann‐Maree Vallence all contributed to the conception and design of the study. Mikaela Frechette completed data acquisition. Jane Tan completed data analysis. All authors contributed to the interpretation of analysis for the work and drafted the work or revised it critically for important intellectual content. All authors have approved the final version of the manuscript, and all authors agree to be accountable for all aspects of the work in ensuring that questions related to the accuracy or integrity of any part of the work are appropriately investigated and resolved. All persons designated as authors qualify for authorship, and all those who qualify for authorship are listed.

## CONFLICT OF INTEREST

None declared.

## Data Availability

The data that support the findings of this study are available from the corresponding author upon reasonable request.
